# High-resolution structure-function mapping of intact hearts reveals altered sympathetic control of infarct border zones

**DOI:** 10.1172/jci.insight.153913

**Published:** 2022-02-08

**Authors:** Ching Zhu, Pradeep S. Rajendran, Peter Hanna, Igor R. Efimov, Guy Salama, Charless C. Fowlkes, Kalyanam Shivkumar

**Affiliations:** 1 Cardiac Arrhythmia Center and Neurocardiology Research Program of Excellence, David Geffen School of Medicine, University of California, Los Angeles, Los Angeles, California, USA.; 2 Department of Biomedical Engineering, George Washington University, Washington, DC, USA.; 3 Department of Medicine, Heart and Vascular Institute, University of Pittsburgh, Pittsburgh, Pennsylvania, USA.; 4 Department of Computer Science, University of California, Irvine, Irvine, California, USA.

**Keywords:** Cardiology, Arrhythmias, Innervation, Mouse models

## Abstract

Remodeling of injured sympathetic nerves on the heart after myocardial infarction (MI) contributes to adverse outcomes such as sudden arrhythmic death, yet the underlying structural mechanisms are poorly understood. We sought to examine microstructural changes on the heart after MI and to directly link these changes with electrical dysfunction. We developed a high-resolution pipeline for anatomically precise alignment of electrical maps with structural myofiber and nerve-fiber maps created by customized computer vision algorithms. Using this integrative approach in a mouse model, we identified distinct structure-function correlates to objectively delineate the infarct border zone, a known source of arrhythmias after MI. During tyramine-induced sympathetic nerve activation, we demonstrated regional patterns of altered electrical conduction aligned directly with altered neuroeffector junction distribution, pointing to potential neural substrates for cardiac arrhythmia. This study establishes a synergistic framework for examining structure-function relationships after MI with microscopic precision that has potential to advance understanding of arrhythmogenic mechanisms.

## Introduction

Myocardial infarction (MI) and its consequent cardiac arrhythmias are leading causes of mortality in the world (1, 2). After MI, injured myocardium creates a substrate for discontinuous electrical propagation (3–5), and concomitant neural remodeling leads to dysregulation at multiple levels of the cardiac autonomic nervous system (6–8). Together, these pathophysiological changes can lead to lethal arrhythmias.

Existing literature on neural control of myocardial impulse propagation has focused on molecular and cellular aspects of cardiomyocyte function in normal and diseased states (9–13). These findings have been vital to the advancement of pharmacologic therapies, which have had significant, though incomplete, success in reducing cardiac morbidity and mortality (14). As antiarrhythmic therapies also focus on structural substrate modification (15), elucidating upstream organ-level neurocardiac control is crucial to bridging the bench-to-bedside gap.

Previous studies using immunostaining of heart sections (16) and, more recently, tissue clearing of whole hearts (17) have shown structural changes in sympathetic nerve fibers innervating the myocardium after MI, with both regions of denervation due to ischemic injury and hyperinnervation due to nerve sprouting. While structural changes after MI have been well described in several animal models (17–20) and in humans (16, 21), their mechanistic significance in arrhythmogenesis remains unclear. Functional studies using multielectrode arrays (18, 19) and optical mapping (22–26) have demonstrated perturbations in impulse propagation with stimulation of the sympathetic nervous system following MI. However, the inability to directly correlate high-resolution structural and functional data from the same heart has impeded our understanding of how structural remodeling of nerves affects functional regulation of the heart after MI.

Our previous work established an important technical basis for high-resolution imaging and semiautomated analysis of global innervation patterns in healthy hearts (27). In this study, we integrate our prior techniques with electrical mapping of intact, diseased hearts to establish a potentially novel, multimodal pipeline allowing direct structure-to-function correlation. By merging functional electrical maps obtained by optical voltage mapping with global structural maps of the heart obtained by tissue clearing, we examine neurocardiac dynamics after MI at microstructural resolution.

## Results

### High-resolution mapping and alignment of cardiac structure with electrical function.

**Figure 1:**
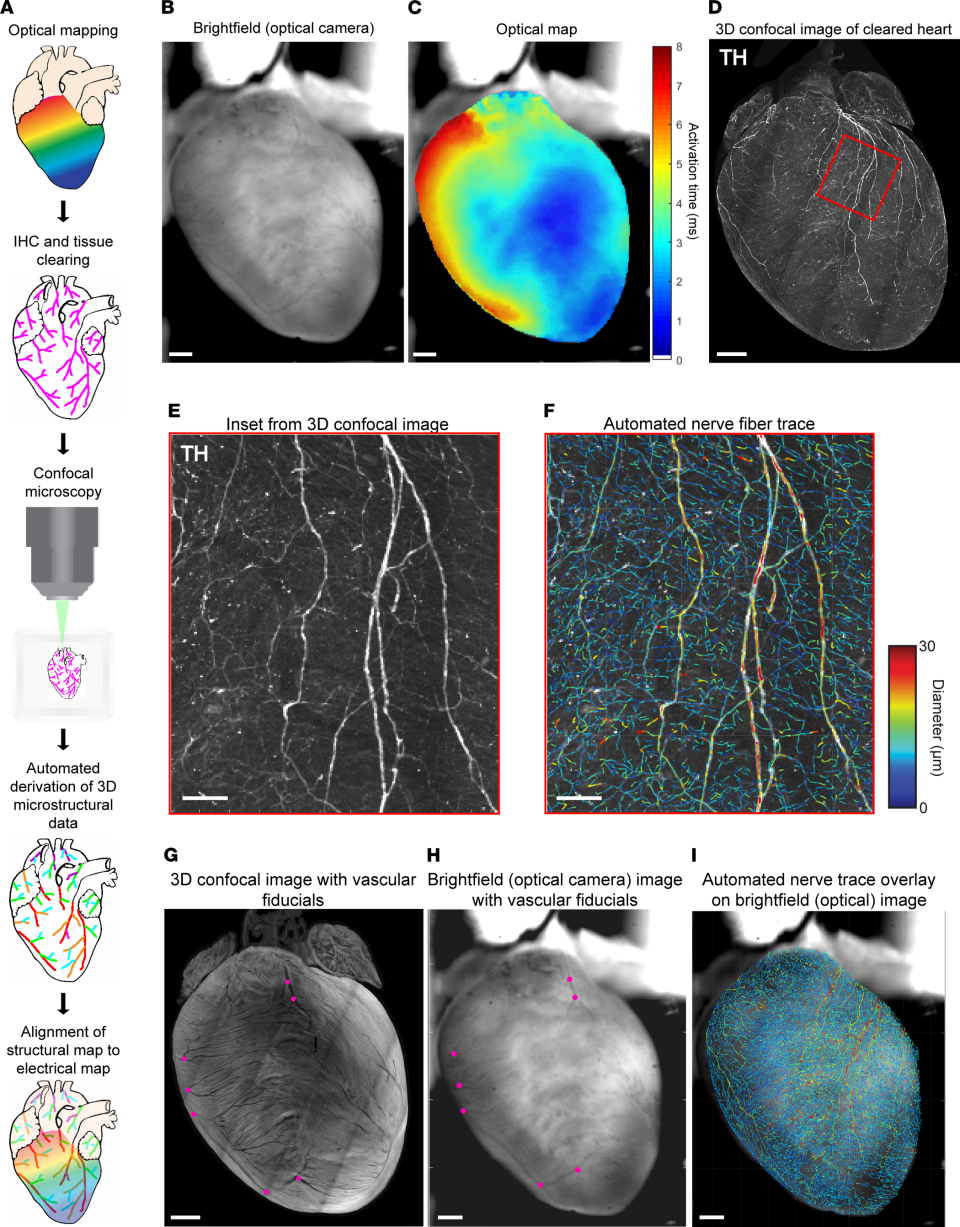
Optical mapping and tissue clearing pipeline to align electrical and structural maps. (**A**) Schematic of optical mapping, clearing, imaging, and automated feature tracing steps in the alignment pipeline. (**B** and **C**) Bright-field image taken simultaneously with optical action potential map showing activation in sinus rhythm. (**D**) Maximum intensity projection (MIP) image of tyrosine hydroxylase–positive (TH-positive) nerve fibers on the ventral surface of the same heart after IHC, tissue clearing, and confocal imaging. (**E** and **F**) High-magnification images of the boxed region in **D**, with TH staining alongside nerve-fiber tracing by computer vision, color-coded by fiber diameter. (**G** and **H**) Venous bifurcations (magenta points) on MIP confocal shell image of a cleared heart alongside bright-field image of same heart were used as fiducial anchors for alignment. (**I**) Automated global nerve-fiber tracing aligned with bright-field image allows spatial correlation with optical action potential data. Scale bars: 1 mm (**B–D** and **G–I**); 100 μm (**E** and **F**).

To evaluate structure-function relationships and changes in these relationships after MI, we developed a pipeline of aligning optical maps of myocardial impulse propagation directly with high-resolution images of myofiber and nerve-fiber structure in the same hearts after tissue clearing and semiautomated fiber tracing (Figure 1A). With a 2-camera system, we first optically mapped action potentials (APs) in normal (sham) hearts and chronic MI hearts and obtained a simultaneous bright-field image of surface vascular features (Figure 1, B and C). We then fixed these hearts, immunolabeled them with the sympathetic nerve marker tyrosine hydroxylase (TH), and performed tissue clearing using a modified, immunolabeling-enabled 3D imaging of solvent-cleared organs (iDISCO) method (28) to allow for high-resolution confocal imaging and semiautomated structural analysis (27) (Figure 1, D–F). To align the structural images with the optical electrical maps, we used vascular fiducial points (venous bifurcations) clearly visible on both the bright-field heart images and the confocal images of muscle autofluorescence (Figure 1, G and H). We found this transformation was sufficient to account for tissue deformation after clearing, bringing fiducials into good alignment over most of the surface visible in the bright-field image. Thus, we were able to overlay structural data, such as global nerve-fiber features (Figure 1I), directly and precisely onto functional electrical data from the same hearts.

### Structure-function alignment precisely correlates global ventricular electrical propagation with myofiber orientation.

**Figure 2:**
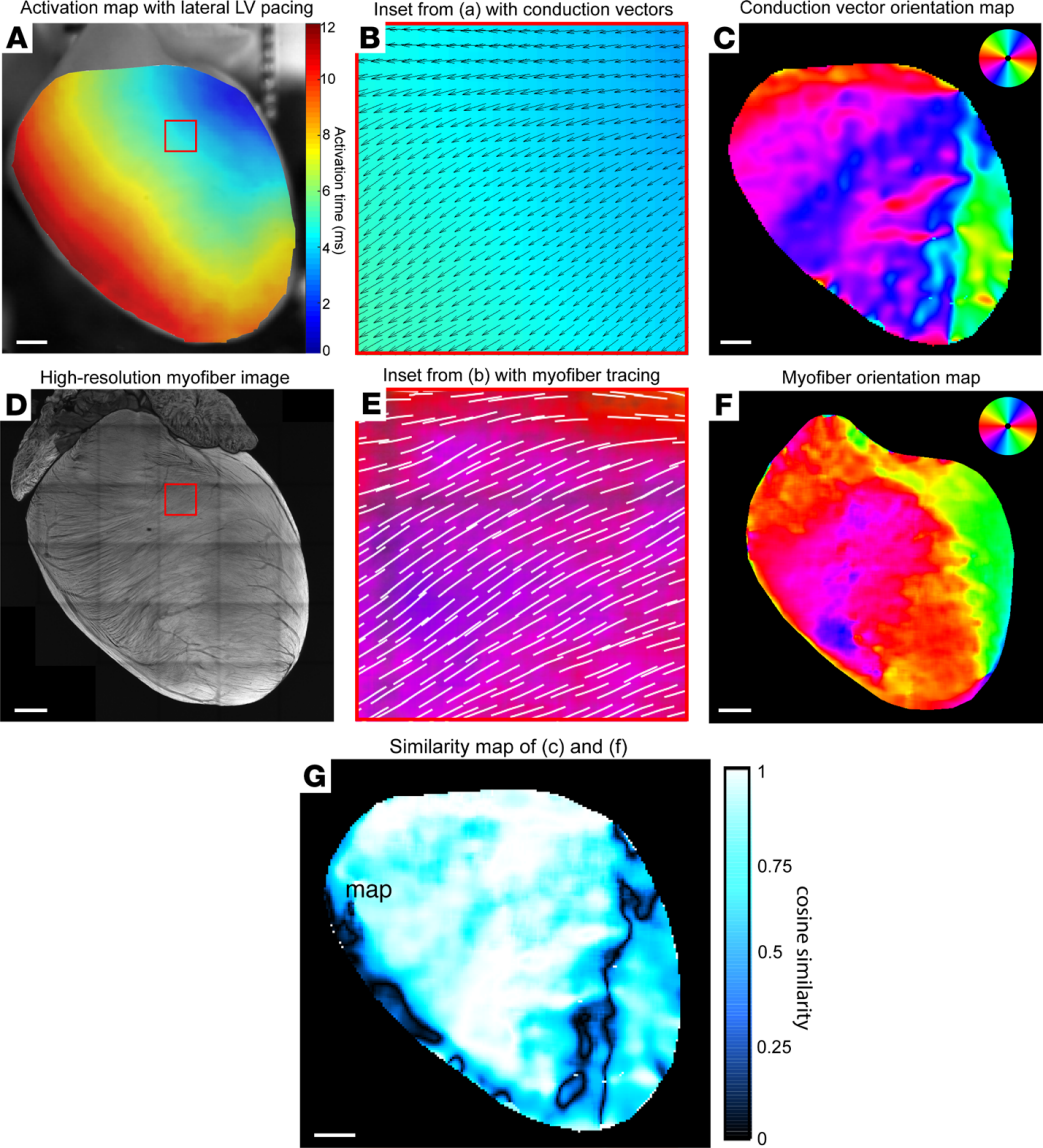
Structure-function alignment correlates global ventricular impulse propagation with myofiber orientation. (**A**) Optical activation map of representative sham heart with basolateral left ventricular (LV) pacing. (**B**) High-magnification image of the boxed region in **A** showing an activation map with overlay of conduction velocity vectors calculated in ElectroMap. (**C**) Global ventricular conduction vector orientation map color-coded by vector angle. (**D**) Maximum intensity projection confocal image of muscle autofluorescence with high-resolution myofiber structure. (**E**) High-magnification image of the boxed region in **D**, with overlay of automated myofiber orientation tracing. (**F**) Global ventricular myofiber orientation map color-coded by fiber angle. (**G**) Cosine similarity map calculated by taking cosine of angular difference between **C** and **F**. Scale bars: 1 mm (**A**, **C**, **D**, **F**, and **G**).

After developing our structure-function alignment pipeline, we validated it in a proof-of-concept analysis to demonstrate the relationship between ventricular myofiber orientation and directionality of electrical propagation in the same heart. Ventricular conduction vectors were calculated from activation maps with basolateral left ventricular (LV) pacing (Figure 2A), exported as angular data matrices (Figure 2B), and visualized as vector orientation color maps (Figure 2C). High-resolution muscle autofluorescence images were used for automated tracing of myofiber orientations (Figure 2, D and E), which were then also visualized as color maps (Figure 2F). We validated this automated myofiber tracing by comparing muscle autofluorescence to viral labeling of cardiomyocytes (Supplemental Figure 1; supplemental material available online with this article; https://doi.org/10.1172/jci.insight.153913DS1).

Once the pairs of functional and structural maps from 4 representative sham hearts were aligned, we first qualitatively assessed the association between myofiber orientation and conduction vector orientation by creating cosine similarity maps (Figure 2G). Of note, our method of alignment detected the area of lowest cosine similarity around the right ventricular (RV) insertion point (where the RV wall attaches to the interventricular septum anteriorly), where there is almost a 90-degree shift in myofiber orientation. Next, we quantitatively analyzed the degree of structure-function concordance in each heart by calculating angular correlation coefficients between myofiber and conduction data matrices and then testing for matrix similarity (Table 1). We found significantly close correlation between structural myofiber orientation and functional conduction vector orientation, thus validating our alignment method and experimentally demonstrating the cable theory of myocardial impulse propagation (5, 29, 30) at the global ventricular level.

### A composite metric of myofiber anisotropy and tissue activation time uniquely defines infarct border zones.

**Figure 3:**
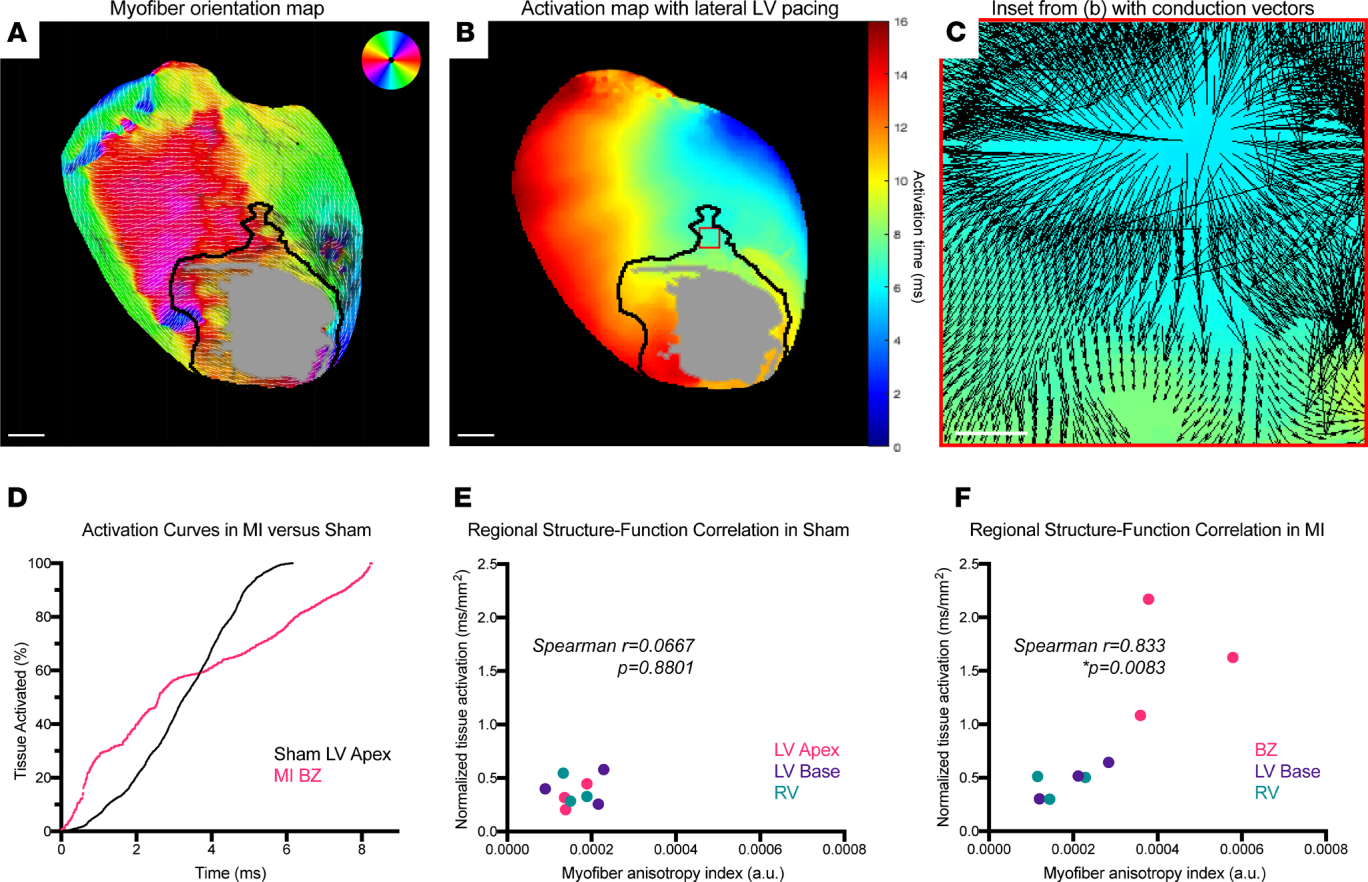
A composite metric of myofiber anisotropy and tissue activation defines infarct border zones. (**A**) Color-coded myofiber orientation map from chronic myocardial infarction (MI) heart with black line delineating border zone (BZ) and gray patch delineating dense scar. Dark patch on left ventricular (LV) lateral wall denotes location of coronary ligature, which was excluded from quantitative analyses. Atria were cropped from image for ease of interpretation. (**B**) Activation map from same chronic MI heart with dense scar region defined by gray patch and BZ delineated by black line. (**C**) High-magnification image of the boxed region in **B**, with activation map with overlay of conduction velocity vectors showing discontinuous propagation. (**D**) Representative tissue activation curves from anatomically defined LV apex region of sham heart (black) versus infarct BZ region (magenta), showing isotropic conduction versus anisotropic and conduction block. (**E** and **F**) Plots of regional myofiber anisotropy indices versus normalized tissue activation times, showing no correlation in sham (Spearman’s *r* = 0.0667, *P* = 0.8801, *n* = 9 regions from 3 mice) versus positive correlation in MI (Spearman’s *r* = 0.833, *P* = 0.0083, *n* = 9 regions from 3 mice). Scale bars: 1 mm (**A** and **B**); 100 μm (**C**).

To begin studying how structural remodeling alters electrical function after MI, we segmented both sham and MI hearts into anatomical regions of interest (ROIs) using specific structural criteria to maintain consistency across hearts. Dense scar and infarct border zone (BZ) were defined using intensity of muscle autofluorescence, and LV basal, LV apical, and RV regions were determined using anatomical landmarks (Supplemental Figures 2–4). Dense scar data were excluded from these analyses due to lack of surviving myocytes (Supplemental Figure 5 and Supplemental Video 1). Quantitative data from these ROIs were extracted from aligned myofiber structure maps (Figure 3A) and activation maps (Figure 3B) and were used to calculate tissue activation times and anisotropy indices of myofiber disorder. Conduction vectors at structurally defined BZ regions displayed discontinuous electrical propagation (Figure 3C). Compared with the isotropic activation curve of sham LV apex, BZ regions displayed activation curves consistent with anisotropic conduction and conduction block (31) (Figure 3D). In plots of myofiber anisotropy index versus tissue activation time, we found that ROIs from sham hearts were all tightly clustered in the low-anisotropy, fast-activation-time region of the plots (Figure 3E, Spearman’s *r* = 0.0667, *P* = 0.8801, *n* = 9 regions from 3 mice). In contrast, the BZs in MI hearts were significantly distinct from other ROIs and localized to the high-anisotropy, slow-activation-time region of the plot (Figure 3F, Spearman’s *r* = 0.833, *P* = 0.0083, *n* = 9 regions from 3 mice). Thus, by integrating regionally specific structural and functional data, we established a quantitative metric that precisely defines the BZ.

### Chronic MI induces altered patterning of ventricular neuroeffector junctions.

**Figure 4:**
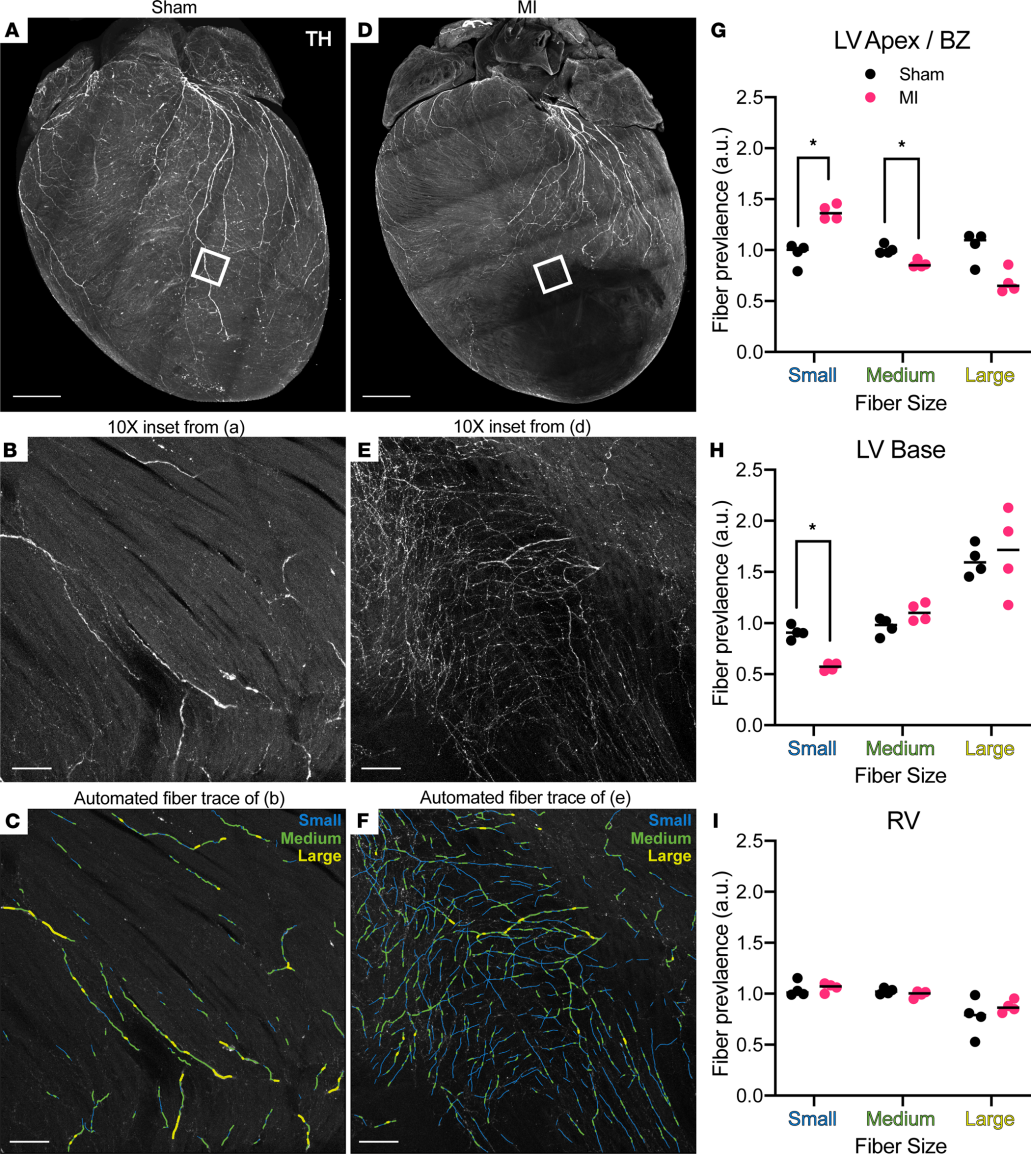
Altered distribution of neuroeffector endings after myocardial infarction. (**A**) Maximum intensity projection (MIP) confocal image of global tyrosine hydroxylase (TH) staining of sham heart (same heart MIP was originally shown in Figure 1D for methodological demonstration purposes). (**B** and **C**) Representative images from boxed region in **A** (original magnification, ×10) from sham left ventricle (LV) showing TH staining and automated fiber tracing, binned by small (1.2–3 μm), medium (3–5 μm), and large (5–100 μm) diameters. (**D**) MIP confocal image of global TH staining in myocardial infarction (MI) heart. (**E** and **F**) Representative images from boxed region in **D** (original magnification, ×10) from border zone (BZ) showing TH staining and automated fiber tracing, binned by small (1.2–3 μm), medium (3–5 μm), and large (5–100 μm) diameters. (**G–I**) Regional comparisons of fiber size prevalence between sham (black) and MI (magenta), with black lines denoting medians and asterisks denoting statistical significance (Mann-Whitney, *P = 0.0286, *n* = 4 mice per group). Scale bars: 1 mm (**A** and **D**); 100 μm (**B**, **C**, **E**, and **F**). RV, right ventricle.

Having established perturbed myocardial structure-function relationships in our chronic MI model, we next turned our attention to assessing sympathetic nerve remodeling. Automatically detected nerve-fiber tracings, from confocal microscopic images of whole-heart TH staining, were binned into small (1.2–3 μm), medium (3–5 μm), and large (5–100 μm) fibers according to previously reported diameters (32–34). The same anatomically segmented ROIs used in the aforementioned myofiber analyses were applied to extract and quantify regional nerve-fiber lengths (Figure 4, A–F). Qualitatively, there was obvious denervation, with absent TH staining at the LV apex (dense scar) of MI hearts compared with sham hearts (Figure 4, A and D). Dense scar data were excluded from these analyses due to the extremely low amount of surviving nerve fibers (Supplemental Figure 5). In both sham and MI hearts, the LV base tended to have significantly more large fibers than medium and small fibers (Kruskal-Wallis, *P* = 0.0048 for sham, *P* = 0.0005 for MI, *n* = 4 mice per group), while the RV had fewer large fibers than medium and small fibers (Kruskal-Wallis, *P* = 0.0132 for sham, *P* = 0.0031 for MI, *n* = 4 mice per group) (Figure 4, H and I).

The changes after MI in small-size fibers were of special interest, as these are both closest to, and include, the neuroeffector varicosities that interface with myocytes to control cardiac function. In MI hearts, the infarct BZ showed a significant increase in small-fiber prevalence compared with sham LV apex (Mann-Whitney, *P* = 0.0286), as well as a decrease in medium-fiber prevalence (Mann-Whitney, *P* = 0.0286) (Figure 4G). This was visually apparent on high-magnification images of BZ compared with the sham LV apex (Figure 4, B and E) and was detectable by our automated fiber tracing algorithm (Figure 4, C and F). Interestingly, the LV base in MI hearts also displayed a decrease in small-fiber prevalence (Mann-Whitney, *P* = 0.0286) compared with sham LV base (Figure 4H). Taken together, these data establish a regional pattern of nerve sprouting at the infarct BZ, along with small-fiber denervation at the remote LV base, specifically indicative of perturbed neuroeffector junction topography.

### Changes after MI in neuroeffector junction patterning underlies regional heterogeneity in sympathetic control of impulse propagation.

Given the altered neuroeffector junction distribution we discovered after chronic MI, we next examined whether these regional neural changes had functional effects on myocardial impulse propagation. Using our alignment technique, we overlaid neural structural data with optical mapping data from the same hearts and assessed regional changes in repolarization after sympathetic stimulation with tyramine, which stimulates norepinephrine release from neuroeffector terminals. For previously discussed reasons, dense scar was excluded from these analyses.

**Figure 5:**
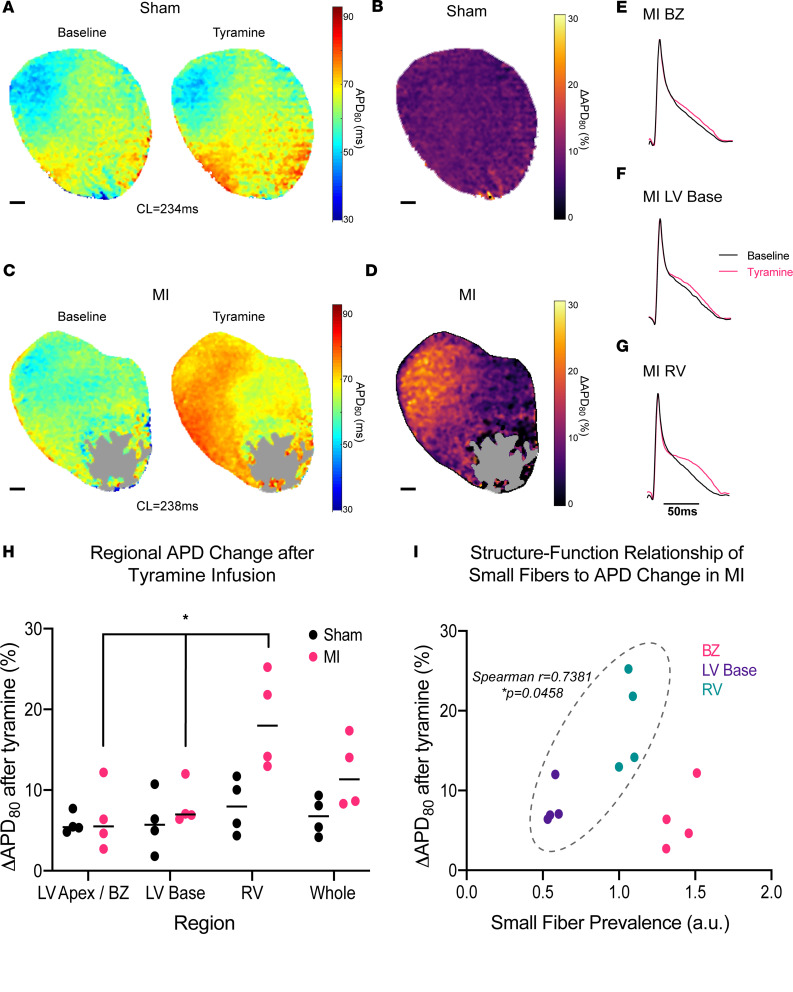
Altered neuroeffector distribution underlies perturbed myocardial sympathetic control after chronic infarction. (**A**) Eighty percent of action potential duration (APD_80_) maps of representative sham heart at baseline and after infusion of 5 μM tyramine. (**B**) Change in APD_80_ (ΔAPD_80_) map of sham heart. (**C**) APD_80_ maps of representative MI heart at baseline and after infusion of 5 μM tyramine. Gray region denotes dense scar. (**D**) APD_80_ map of MI heart. (**E–G**) Representative action potentials at baseline (black) and after tyramine (magenta) in anatomically segmented regions of MI heart. (**H**) Comparison of regional, tyramine-mediated changes in APD_80_ between sham and MI hearts, with MI hearts showing significant regional variation in tyramine effect (Kruskal-Wallis, *P = 0.0132, *n* = 4 mice per group) while sham hearts showed no significant regional variation (Kruskal Wallis, *P* = 0.7463, *n* = 4 mice per group). (**I**) Plot of regional small-fiber prevalence in MI hearts versus tyramine-mediated APD change, with positive correlation in left ventricular (LV) base and right ventricular (RV) regions (Spearman’s *r* = 0.7381, *P* = 0.0458, *n* = 8 regions from 4 mice) but no correlation when border zone (BZ) is included (Spearman’s *r* = 0.021, *P* = 0.956, *n* = 12 regions from 4 mice). Scale bars: 1 mm (**A–D**).

We found that in sham hearts tyramine infusion caused an expected initial prolongation of 80 percent of AP duration (APD_80_) (35) in an evenly distributed fashion across the whole heart (Kruskal-Wallis, *P* = 0.7463) (Figure 5, A, B, and H). In contrast, MI hearts exhibited significant regional variation in APD_80_ prolongation after tyramine infusion (Kruskal-Wallis, *P* = 0.0132) (Figure 5, C, D, and H). Specifically, there was more APD_80_ prolongation of the RV in MI hearts compared with the LV base and infarct BZ (Mann-Whitney *P* = 0.0286, *n* = 4 mice per group) (Figure 5, E–H). When we correlated small-fiber distribution after MI with these functional repolarization changes (Figure 5I), we found that, while there was a positive correlation between small-fiber prevalence and tyramine-induced APD_80_ prolongation at the LV base and RV regions (Spearman’s *r* = 0.7381, *P* = 0.0458), the BZ notably lacked this functional correlation, despite having the highest prevalence of small fibers (Spearman’s *r* = 0.021, *P* = 0.956). These data demonstrate a direct, anatomically precise relationship between regional small-fiber content — a surrogate index of neuroeffector junction quantity — and sympathetic control of myocardial repolarization, with the interesting exception of the functionally distinct infarct BZ.

## Discussion

We developed a high-resolution platform for precisely aligning functional maps of electrical propagation to structural maps of neurocardiac remodeling after MI, using optical mapping, state-of-the-art intact-heart imaging, and computer vision algorithms for semiautomated feature detection. Using this platform, we report several potentially novel findings: (a) direct spatial correlation of ventricular myofiber structure to directionality of AP propagation at the global ventricular level; (b) a mathematically precise definition of the infarct BZ that integrates both its distinctive microstructural and functional features; (c) perturbed neuroeffector-junction topography of the whole ventricle after MI; and (d) a direct relationship between neuroeffector-junction distribution after MI and altered sympathetic control of impulse propagation.

Our structure-function alignment method demonstrates close, global concordance between myofiber architecture and conduction vector fields for the first time to our knowledge in intact ventricles, a relationship which had previously only been studied at the single-myocyte level (29, 36) or through computational modeling of myocardial function (37–39). The high spatial resolution of our alignment pipeline also allows structurally precise regional analyses, which we utilized to define the infarct BZ with a potentially novel composite metric that encompasses both myofiber anisotropy as well as discontinuous impulse propagation. The relationship between myofiber disorder and conduction block has been demonstrated previously in computational models (38) and low-resolution electrode recordings or optical maps from grossly approximated BZ regions (19, 40–43), but these studies utilized methods of localizing the BZ that are highly variable and subjective. Our findings represent the first experimental correlation of perturbed myofiber architecture to disordered electrical propagation at this degree of microstructural resolution and mathematical precision to our knowledge. This integrative approach to defining the BZ region by both structure and function offers unparalleled anatomical consistency for studies of its pathophysiology.

Previous studies examining the distribution after MI of cardiac sympathetic innervation relied on manual quantification of total nerve immunofluorescence (17, 44–46) and thus lacked specificity for nerve endings versus larger pass-through fibers. In contrast, our microstructural feature detection algorithms identified regional patterns of size-specific nerve-fiber remodeling, allowing focus on the functionally important neuroeffector junction. Because we were able to automatically detect and define small-fiber dimensions specifically by the size of sympathetic neuroeffector terminals (32–34), we revealed the possibly novel and important finding of small-fiber predominance at the infarct BZ and small-fiber decrease at the remote LV base. This perturbation after MI of small-fiber topography suggests an altered neural-myocardial interface, with regional loss of neuroeffector terminals at the LV base and nerve sprouting at the BZ.

Moreover, we discovered that the altered small nerve-fiber pattern after MI has a direct relationship to altered sympathetic control of ventricular repolarization. Specifically, we found that chronic MI hearts display a correlation between regional variation in tyramine-mediated APD prolongation (higher in RV compared with LV base) and the spatial distribution of small-fiber prevalence (also higher in RV compared with LV base). This finding is especially important, as it establishes a potential neural-structural substrate for the sympathetically driven increase in regional heterogeneity of repolarization, which may lead to arrhythmogenic gradients (5, 47).

Interestingly, the infarct BZ did not display higher APD prolongation compared with other regions, despite the higher small-fiber prevalence suggestive of nerve sprouting. While nerve sprouting has previously been shown to be localized at the infarct BZ and to correlate with sudden death (20, 48), the precise pathophysiological processes remain unclear. Several possible mechanisms may underlie our finding. These sprouts may be dysfunctional in tyramine uptake via the norepinephrine transporter (49), which has been previously shown to be downregulated after MI (44, 49). Alternatively, the sprouts may have altered neurotransmitter release functions (45), or the cardiomyocytes in this region may have altered adrenergic receptor profiles (39, 50). That nerve sprouting at the BZ does not align with tyramine-mediated influence on APD points to the functional distinctiveness of this boundary between surviving myocardium and dense scar and promotes the generation of highly specific hypotheses regarding sympathetically driven arrhythmias after MI.

Taken together, these data generated from our platform for structure-function alignment establish an important framework for understanding how structural cardiac diseases such as MI perturb specific myocardial electrical functions, as well as the neural substrates and mechanisms that control these functions. While most of the existing neurocardiac literature focuses on molecular and cellular alterations in arrhythmogenic heart disease (9–11, 39, 50, 51), current clinical therapies for arrhythmia actually depend heavily on anatomical substrate modification (15) and neuromodulation at multiple structures of the autonomic nervous system (52). These therapies have benefited greatly from advancements in clinical imaging (53) to localize potential arrhythmogenic substrates, yet spatially correlating these substrates with their functional roles remains an important challenge. Thus, our study addresses the crucial need to understand neurocardiac dynamics after MI at the whole-organ level, while still offering the high spatial resolution necessary to target the microstructural features underlying arrhythmogenic processes.

Overall, the synergistic neural-myocardial framework we present in this study is vitally important to elucidating the pathophysiology leading to sudden cardiac death. Our approach to structure-function alignment could feasibly incorporate emerging technologies, such as spatial detection of interstitial neurotransmitter levels with fast-scanning cyclic voltammetry (54) and optical norepinephrine tracers (55), to generate additional mechanistic insights. Ultimately, a combination of such techniques will be needed to enable the development of more powerful and targeted neuromodulatory therapies for heart disease.

## Methods

### Animals.

All mice used were male, from the C57BL/6J strain, and obtained from The Jackson Laboratory. Survival surgeries to create chronic MI were performed when mice were 12 weeks (±5 days) of age (weighing 22–28 g), and terminal optical mapping experiments occurred approximately 4 weeks after MI, when mice were 16 weeks (±4 days) of age.

### Creation of chronic MI mouse model.

Mice were anesthetized with isoflurane (2%), endotracheally intubated, and mechanically ventilated. A small thoracotomy incision was made in the left 7th or 8th intercostal space to access the heart, the pericardium was opened with fine forceps, and the left coronary artery (analogous to the human left anterior descending artery) (56) was ligated with 8-0 silk suture at the midlevel of the LV. Acute transmural ischemia was confirmed by visualization of myocardial blanching and ST elevation on electrocardiogram. The incision was then closed in 2 layers (muscle and skin), and the animal was extubated and allowed to recover on a temperature-controlled surface. Carprofen (5 mg/kg, intraperitoneal injection every 24 hours) and buprenorphine (0.02 mg/kg, intraperitoneal injection every 8 hours) were given for pain control on the day of and for 48 hours after surgery. Sham surgeries included all steps except coronary artery ligation.

### Optical mapping of APs.

In Langendorff-perfused hearts, optical mapping of V_m_ was performed as previously described (57, 58). Briefly, mice were sacrificed per protocol by anesthesia with 5% isoflurane followed by cervical dislocation. Hearts were removed immediately and perfused via the aortic root with Tyrode’s solution (130 mM NaCl, 1.25 mM CaCl_2_, 5 mM KCl, 1.2 mM NaH_2_PO_4_, 1.1 mM MgCl_2_, 22 mM NaHCO_3_, and 50 mM dextrose). Hearts were immobilized and immersed in a Tyrode’s solution bath within a 3D-printed chamber to reduce motion artifact. Perfusate and bath temperature were maintained at 36.6°C–37°C. Hearts were stained with bolus injections of voltage-sensitive dye RH237 (8–10 μl of 2 mg/ml in DMSO, Thermo Fisher Scientific, S1109) into the coronary perfusate. Blebbistatin (Cayman Chemical, 13186) was added to the perfusate at a concentration of 1.7 μg/mL for excitation-contraction uncoupling.

Light from 2 collimated ultra-high-power LED (Prizmatix, UHP-T-520-EP) guides was focused on the ventral epicardial surface of the heart for excitation. Emitted fluorescence was collected using a tandem-lens arrangement of Nikon NIKKOR 50 mm f/1.2 camera lenses and split with a 635 nm dichroic mirror (Edmund Optics, 87064) (59). The V_m_ signal was filtered at 690 ± 50 nm (Chroma ET690/50m), and a simultaneous bright-field image for vascular visualization and alignment was taken using the shorter-wavelength filtered light at 590 ± 33 nm (Chroma ET590/33m). The emitted Vm signals and bright-field images (for vascular alignment) were recorded using 2 CMOS cameras (SciMedia, MiCAM N256) with a sampling rate of 1.03 kHz and 256 × 256 pixels with a 14 × 14 mm field of view. Pixel resolution of the images was approximately 55 × 55 μm. Data were acquired in 2-second intervals before and after addition of tyramine to the perfusate at a concentration of 5 μM. Data acquisition was done using BV Workbench software version 1.7.10 (SciMedia).

For conduction velocity analyses, epicardial pacing was performed from the basolateral LV wall at a cycle length (CL) of 167 ms (with current of 1.1–1.3 mA and pulse width of 0.8 ms), using a Transonic Scisense 1.1F mouse EP catheter (FTS-1113A-0518). For analyses of tyramine effect on repolarization, time points before and after tyramine were taken in sinus rhythm, just before heart rate increase, to allow comparison at the same CL (Supplemental Figure 6).

### Optical mapping data analysis.

Optical mapping data were analyzed using the open-source software ElectroMap (31). V_m_ activation maps were displayed as isochronal maps generated from points of maximum upstroke (d*F*/d*t*)_max_ as well as depolarization midpoint of optical APs. Repolarization maps were generated from points of APD_80_. A minimum of 4 beats was averaged at baseline and after tyramine infusion. A 3 × 3 Gaussian spatial filter, Top-hat, and Savitzky-Golay filters were applied to correct for baseline drift and noise. Maps were exported as 256 × 256 data matrices for alignment with structural data and quantitative analyses.

### IHC and tissue clearing.

After optical mapping, whole mouse hearts were fixed by immersion in 4% paraformaldehyde/PBS overnight at 4°C and then washed 3 times for 1 hour in 0.01 M PBS at room temperature. Hearts were stained and cleared using a modified iDISCO protocol (28). Fixed hearts were dehydrated by graded methanol treatments (20%, 40%, 60%, and 80% methanol in H_2_O [vol/vol], each for 1 hour at room temperature), washed twice with 100% methanol for 1 hour at room temperature, and chilled at 4°C. Hearts were then immersed in 66% dichloromethane/33% methanol overnight at room temperature with agitation, washed twice in 100% methanol for 1 hour at room temperature, and chilled to 4°C. Next, hearts were bleached with 5% H_2_O_2_ in methanol (vol/vol) overnight at 4°C. After bleaching, hearts were rehydrated with graded methanol treatments, followed by one wash with 0.01 M PBS and 2 washes with 0.01 M PBS with 0.2% Triton X-100, each for 1 hour at room temperature. Hearts were permeabilized with 0.01 M PBS with 0.2% Triton X-100, 20% DMSO, and 0.3 M glycine and blocked with 0.01 M PBS with 0.2% Triton X-100, 10% DMSO, and 5% normal donkey serum, each for 2 days at 37°C with agitation. Hearts were incubated in sheep anti-TH (EMD Millipore, AB1542, 1:200) and/or rabbit anti-periostin (Abcam, ab14041, 1:200) diluted in 0.01 M PBS with 0.2% Tween-20 and 10 mg/ml heparin (PTwH) for 5–7 days at 37°C with agitation. Hearts were then washed 4–5 times in PTwH overnight at room temperature before incubation in donkey anti-rabbit Cy3 (Jackson ImmunoResearch, 711-165-152, 1:300) and/or donkey anti-sheep Alexa Fluor 647 (Jackson ImmunoResearch, 713-605-147, 1:300) secondary antibodies diluted in PTwH for 5–7 days at 37°C with agitation. Primary and secondary antibodies were replenished approximately halfway through incubation period. Hearts were then washed several times in PTwH overnight at room temperature. For clearing, stained hearts were dehydrated with a graded methanol series and incubated in 66% dichloromethane/33% methanol for 3 hours at room temperature with agitation. Hearts were then washed twice in 100% dichloromethane for 15 minutes at room temperature. Hearts were stored in benzyl ether (MilliporeSigma, 108014 ALDRICH; refractive index, 1.55) for up to 7 days prior to imaging.

### Confocal imaging.

Hearts were mounted in benzyl ether with adhesive plastic spacers (Sunjin Labs, IS012 and IS012). Images were acquired on a confocal laser scanning microscope (Zeiss, LSM 880) fitted with the following objectives: Fluar 5×/0.25 M27 Plan-Apochromat (working distance, 12.5 mm) and 10×/0.45 M27 (working distance, 2.0 mm). Images were taken at both ×5 and ×10 magnifications for specific ROIs, such as RV and LV base, prior to the whole hearts being imaged at ×5 in tiles with *xy* resolution of 1.661 μm and *z* resolution of 8.29 μm.

### Image processing and automated structural mapping.

All image processing was performed using Zeiss Zen 2.1 v11, ImageJ (NIH), Fiji (60), and custom Matlab scripts (available upon request from corresponding author). Computational tracing of nerve fibers was performed using a customized version of the open-source software neuTube (61). neuTube software was originally developed for tracing morphology of single cells. Tracing nerves in large-image volumes required additional pre- and postprocessing, including (a) partitioning large volumes into smaller tiles, tracing each tile, and reassembling the traced morphologies and (b) filtering out spurious junctions between parallel fibers and inaccurate fiber diameter estimates arising due to background staining. To quantify myofiber orientation distributions, we utilized confocal images of muscle autofluorescence. This was validated using comparison of autofluorescence with virally labeled myocyte imaging (Supplemental Figure 1). We computed the image gradient orientation at each point and then smoothed the gradient orientation field using a Gaussian-weighted moving average window of size of σ = 100 μm.

Structural images were aligned to functional images using vascular fiducial points from bright-field images obtained during optical mapping. For each sample, 5–10 fiducial points (branches in vasculature, sutures, or scars) visible in both bright-field optical mapping and confocal images were used to fit a perspective warping (homography) between the 2 images. Only structural data from the outer 100 μm thick “shell” of each heart were used for alignment and correlation with optical mapping data. This depth was determined empirically by light penetration experiments (Supplemental Figure 7).

### Quantitative data analysis.

Conduction velocity and activation curves were calculated using ElectroMap. Regional myofiber anisotropy is represented as a normalized index defined as the coefficient of variation (angular standard deviation over the angular mean) of fiber angles, divided by the total surface area of the segmented ROI: 

  Equation 1.
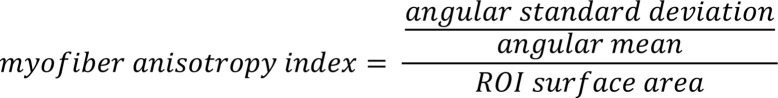


Per prior reports (32–34), nerve-fiber size bins were defined by the following diameters: small fibers were 1.2–3 μm, medium fibers were 3–5 μm, and large fibers were 5–100 μm. 1.2 μm was used as the lower limit of small fibers to minimize detection of nonspecific background staining. Fiber prevalence is represented as a normalized index defined as the proportion of a particular size fiber in a ROI, divided by the proportion of that fiber size in the whole heart: 

  Equation 2.
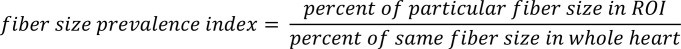


Cosine similarity maps were generated by calculating the cosine of the difference in angles at each pixel between the functional (conduction velocity) maps and the structural (myofiber orientation) maps.

### Statistics.

Angular correlation coefficients and matrix similarity *P* values were calculated using open-source Matlab scripts for circular statistics (62) and a customized Matlab script based on open-source code utilizing Mantel’s matrix similarity test (BRAMILA pipeline v2.0, available at https://version.aalto.fi/gitlab/BML/bramila/-/blob/f0e40fba79744c875d6eaa7f39c5b9aafa6bcba9/bramila_mantel.m) using 1000 permutations. Data are presented as medians in figures, and sample sizes are indicated in figure legends or main text. All statistical analyses for comparison are indicated in figure legends or main text, were 2 tailed, and were performed in Prism 9.0.2 (GraphPad). Kruskal-Wallis, Mann-Whitney, Spearman’s r were used for statistical testing. *P* values of less than 0.05 were considered significant.

### Study approval.

Animal experiments complied with all relevant ethical regulations and institutional regulations of the UCLA Animal Research Committee, which approved these studies (protocol 16-033).

## Author contributions

CZ, PSR, PH, CCF, and KS designed the study. CZ, PSR, and PH developed and validated the technical bases for performing the mapping studies, with guidance from IRE and GS. CZ performed the animal surgeries, tissue collection, optical mapping, IHC and tissue clearing, confocal imaging, and optical data analyses. CCF performed image processing, aligned structural and functional maps, and developed computational algorithms for semiautomated structural analyses. CZ and CCF performed quantitative analyses for structure-function data correlation. CZ prepared the figures. CZ wrote the manuscript, with assistance from PSR and PH. All authors contributed to the final version of the manuscript.

## Supplementary Material

Supplemental data

Supplemental video 1

## Figures and Tables

**Table 1:**
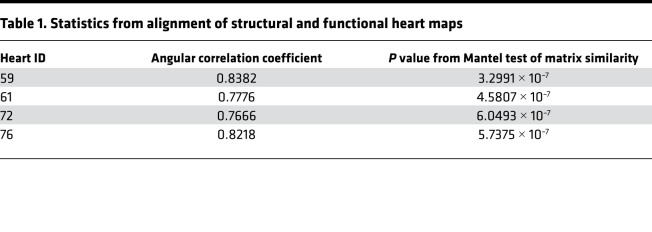
Statistics from alignment of structural and functional heart maps

## Version 1 02/08/2022

Electronic publication
