# Supplementary material for: High-resolution structure-function mapping of intact hearts reveals altered sympathetic control of infarct border zones
Source: JCI Insight. 2022 Feb 8;7(3):e153913. doi: 10.1172/jci.insight.153913 (PMC8855798; doi:10.1172/jci.insight.153913)

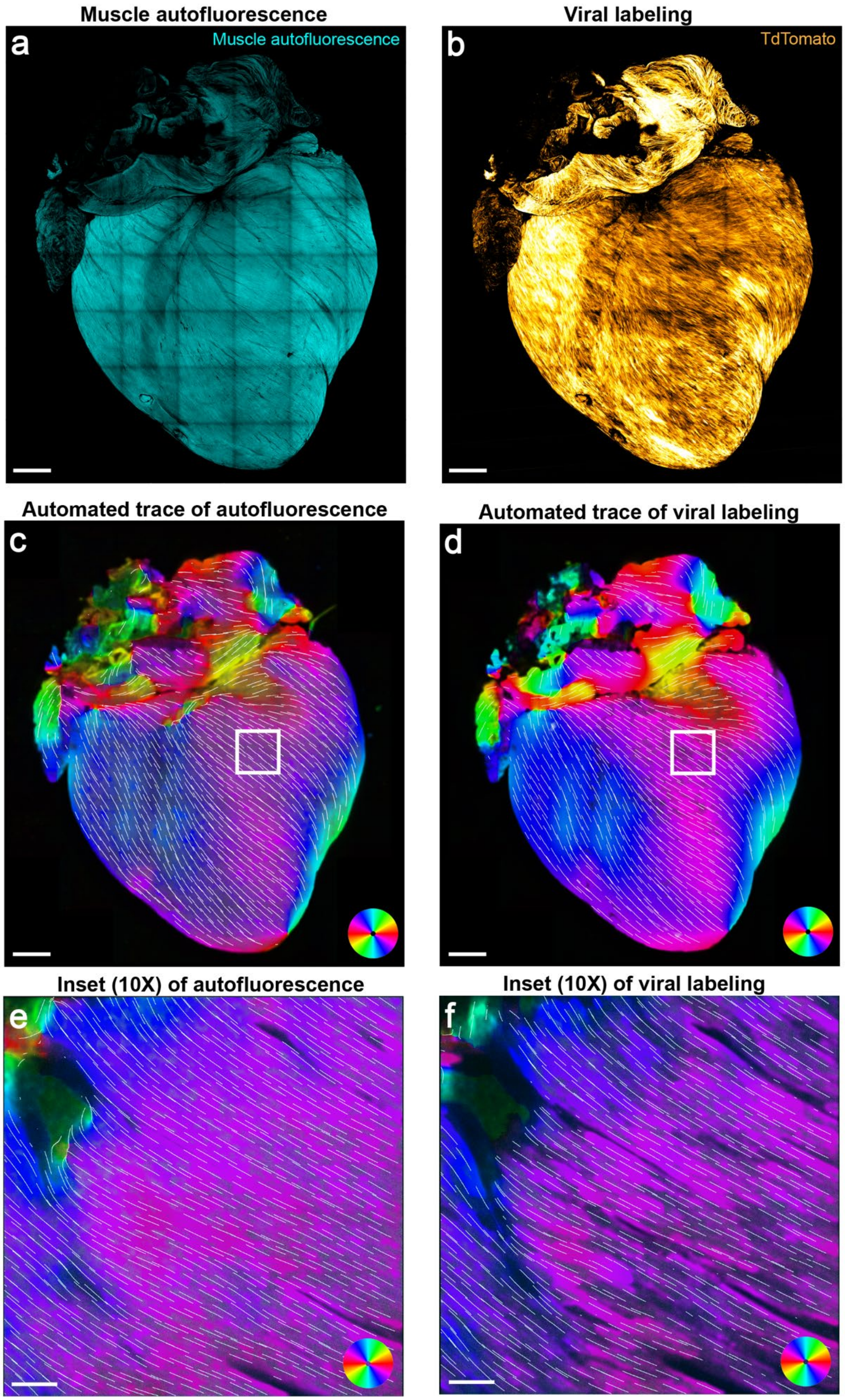

Supplemental Figure 1. Validation of using muscle autofluorescence to trace myofiber orientation. Muscle autofluorescence was imaged by excitation at 488nm and emission window collected from 491-554nm **(a)**. This was compared to fluorescent viral labeling of the same heart muscle **(b)** which was done by retro-orbital injection of AAV-PHP.S-CAG-TdTomato (1x10^{12} vg, Addgene, #59462-PHP.S), followed by confocal

imaging after 2 weeks with excitation at 561nm and emission window from 562-625nm. Automated tracing of myofiber orientation was done for both autofluorescence and viral labeling images **(c-f)**, and Mantel matrix similarity testing was done for 2 representative hearts (angular correlation coefficients 0.9281 and 0.9169, $p=1.293 \times 10^{-9}$ and 3.127×10^{-8}, 1000 permutations). All images are maximum intensity projections. Scale bars = 1mm (**a-d**) and 100µm (**e-f**).

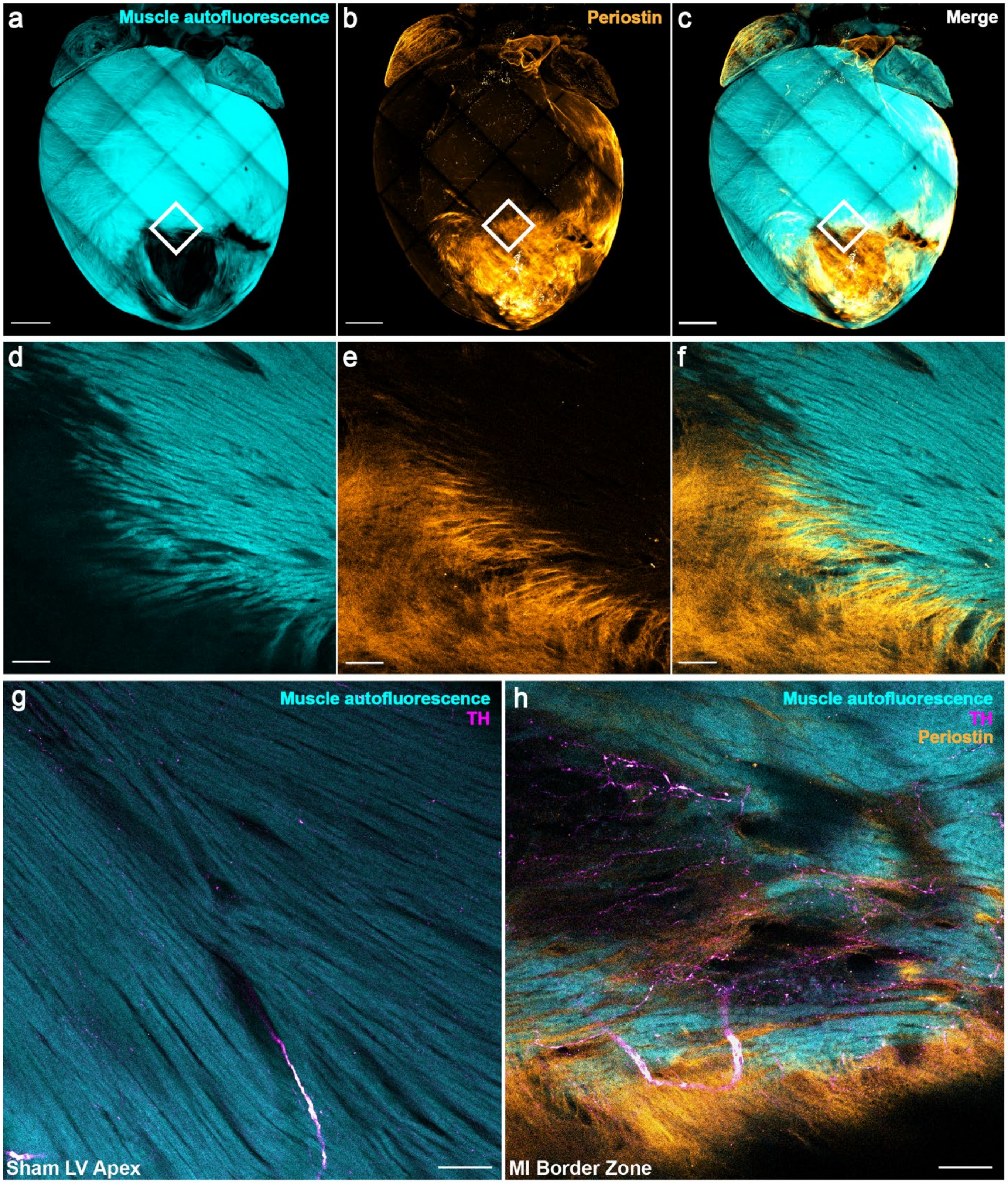

Supplemental Figure 2. Validation of using autofluorescence to define dense scar versus surviving myocardium. Periostin (1:200, abcam, ab14041) staining of cleared chronic myocardial infarction heart imaged at 5X magnification **(a-c)** shows dense scar is visibly devoid of muscle autofluorescence. Zoomed inset of the border zone (BZ) (10X magnification) **(d-f)** shows interdigitation of periostin-labeled scar with living muscle. In contrast to normal left ventricular apex **(g)**, infarct BZ at the interface of periostin with muscle autofluorescence demonstrates nerve sprouting **(h)**. All images are maximum intensity projections. TH = tyrosine hydroxylase. Scale bars are 1mm (**a-c**) and 100µm (**d-h**).

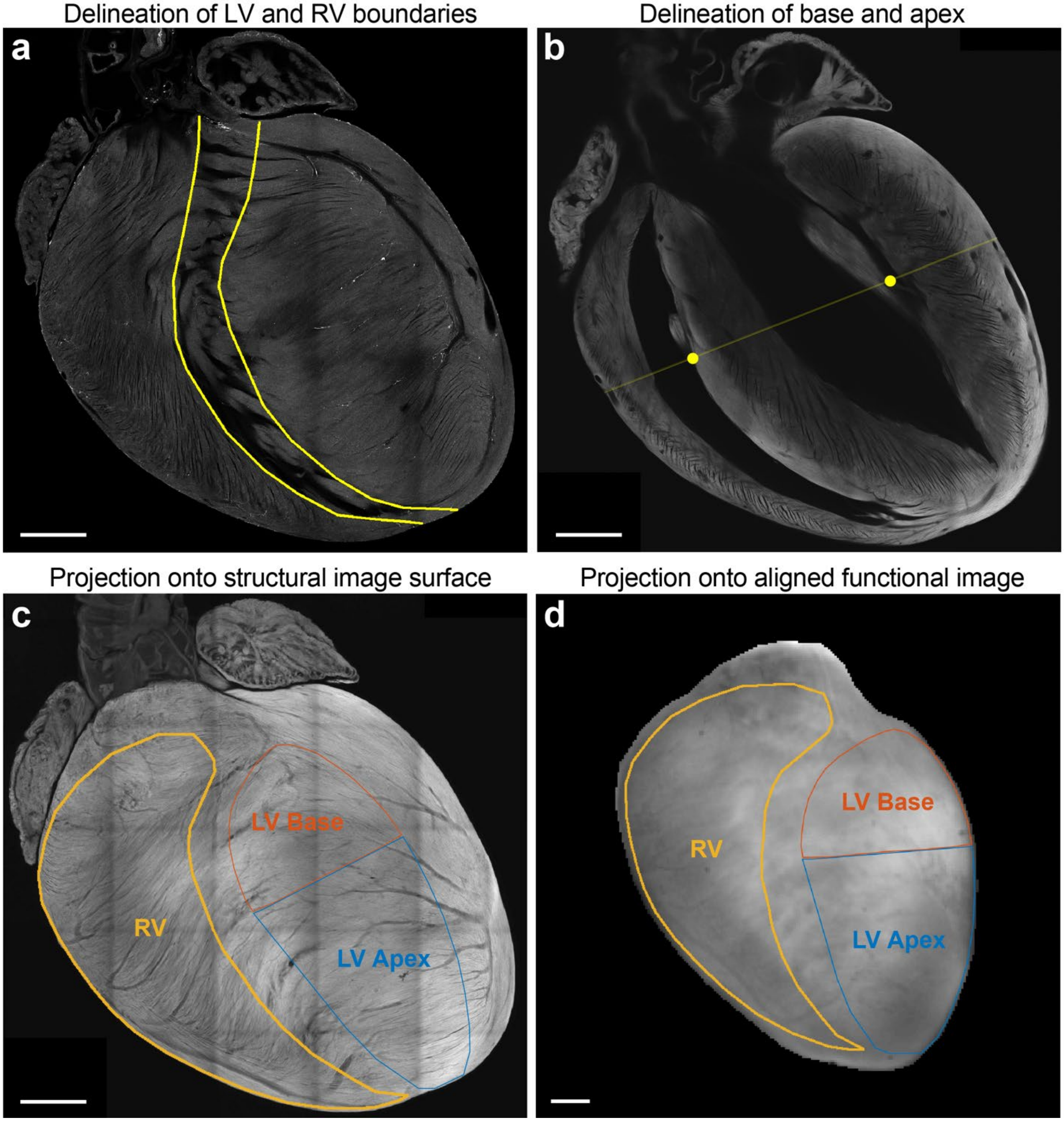

Supplemental Figure 3. Defining regions of interest (ROIs) based on 3-dimensional anatomical landmarks. For consistency across hearts, ROIs were defined by anatomical landmarks in confocal microscopic image stacks of muscle autofluorescence in cleared hearts. Left ventricular (LV) and right ventricular (RV) boundaries were carefully delineated by exclusion of the ventricular septal trabeculations **(a)**. LV base and apex were delineated by drawing a line between two points: the RV septal papillary-muscle-chordae-tendinae junction and the LV anterolateral papillary-chordae junction **(b)**. All myocardial infarction border zone and dense scar regions in analyzed hearts were visually confirmed to be below this line. These delineations were then projected onto the structural image surface of the heart **(c)**, which, when aligned by vascular fiducials with the brightfield optical image **(d)**, allowed projection of the ROIs onto the functional electrical maps. Scale bars are all 1mm.

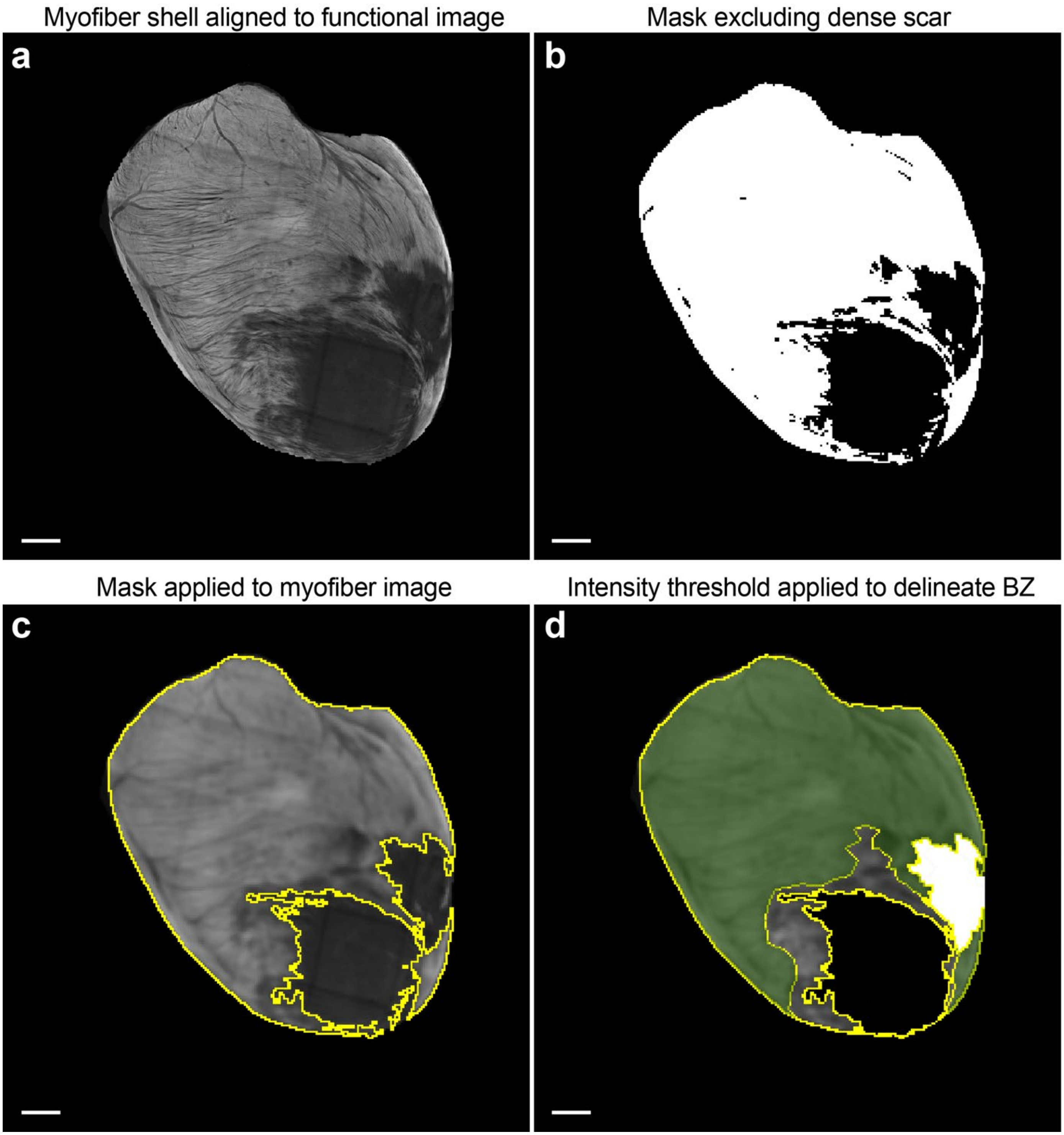

Supplemental Figure 4. Defining border zone and dense scar based on myofiber imaging. To maintain structural consistency in border zone (BZ) definition across infarcted hearts, 16-bit maximum intensity projection images of 100μm-thick epicardial shells were created **(a)**, and a mask was applied to define regions with <100μm-thick surviving myocardium as dense scar **(b)**. A 2x2 pixel Gaussian blur was then applied to the remaining surviving myocardial image **(c)**, and a threshold was applied at half the maximal fluorescence intensity of the image to define the BZ. Panel **(d)** shows the resulting segmentation, where the region in green is surviving myocardium above the fluorescence intensity threshold, the region in gray is below the threshold and thus defined as BZ, the dense scar is in black, and the location of the coronary ligature is in white (also excluded from quantitative analyses). All scale bars are 1mm.

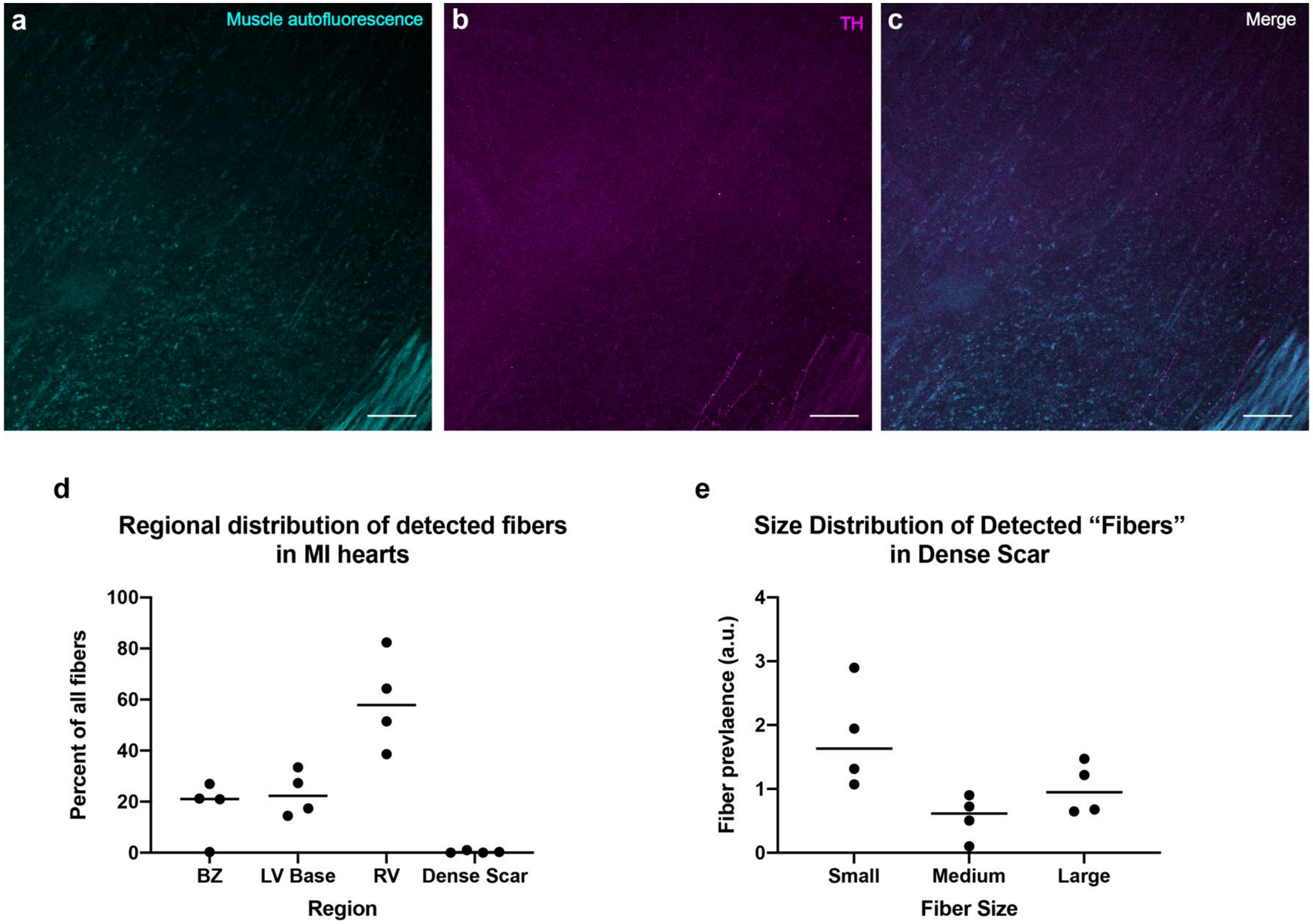

Supplemental Figure 5. Dense scar structural data. We excluded regions of dense scar as defined in Supplemental Figure 4 from our quantitative structural analyses due to the negligible amount of living muscle and nerve fibers detected in these regions, as shown qualitatively by immunofluorescence in 4 cleared infarcted hearts **(a-b)** and quantitatively by automated nerve fiber detection **(d)**. The relative predominance of "small fibers" in dense scar **(e)** possibly reflects detection of background or artifactual signal. Scale bars in **(a-c)** are 100μm. TH=tyrosine hydroxylase. a.u. = arbitrary units.

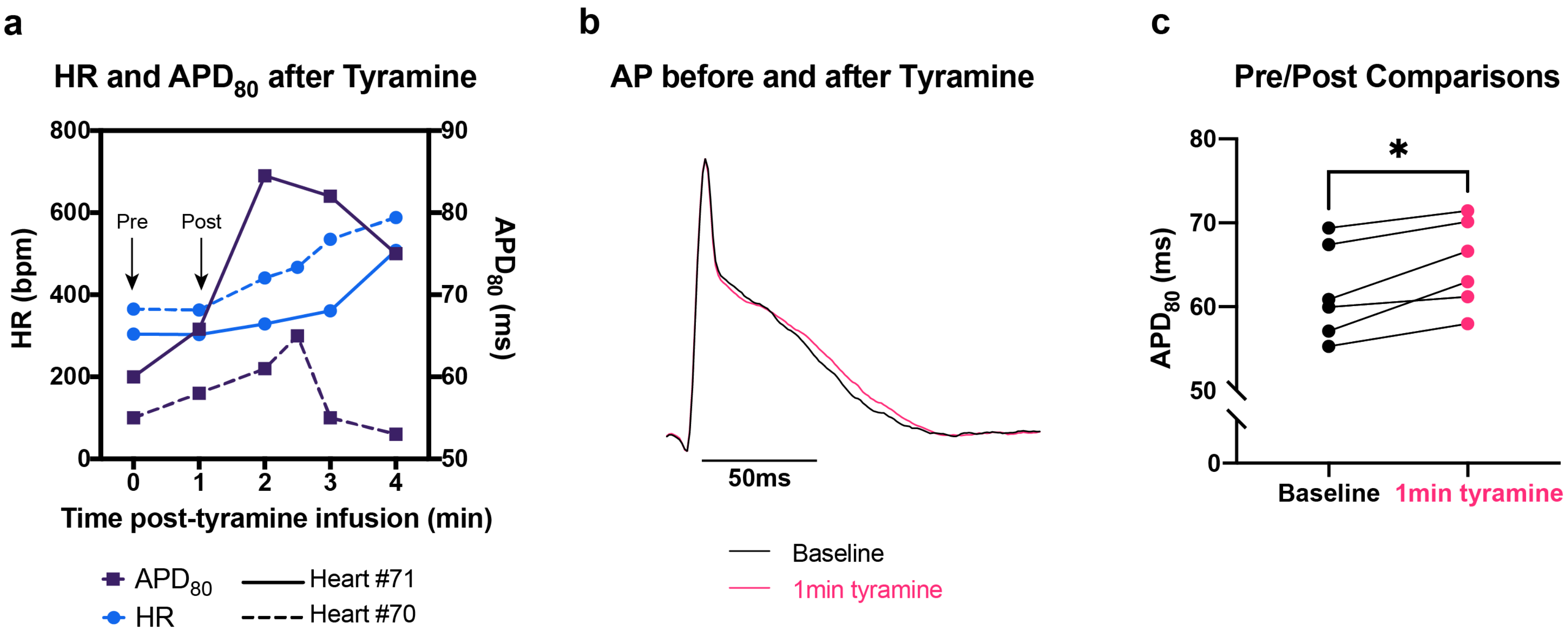

Supplemental Figure 6. Validating choice of pre- and post- tyramine time points for action potential duration (APD) analyses. To avoid potentially confounding neuromodulatory effects of pacing, we chose to analyze tyramine-mediated APD changes in sinus rhythm. We found that in all our ventricular APD maps, there was a global increase in 80% of APD (APD_{80}) prior to an increase in heart rate (HR), at approximately 1 minute after 5 μM tyramine infusion, with representative data shown in **(a)** and **(b)**. This increase suggests that tyramine has a myocardial effect prior to its nodal effect and was statistically significant across all the sham hearts we mapped for this study **(c)** (Wilcoxon $p=0.0312$, $n=6$ mice). Thus we defined pre- and post- tyramine timepoints of APD analyses as immediately before, and approximately 1 minute following, the start of tyramine infusion, just prior to HR increase.

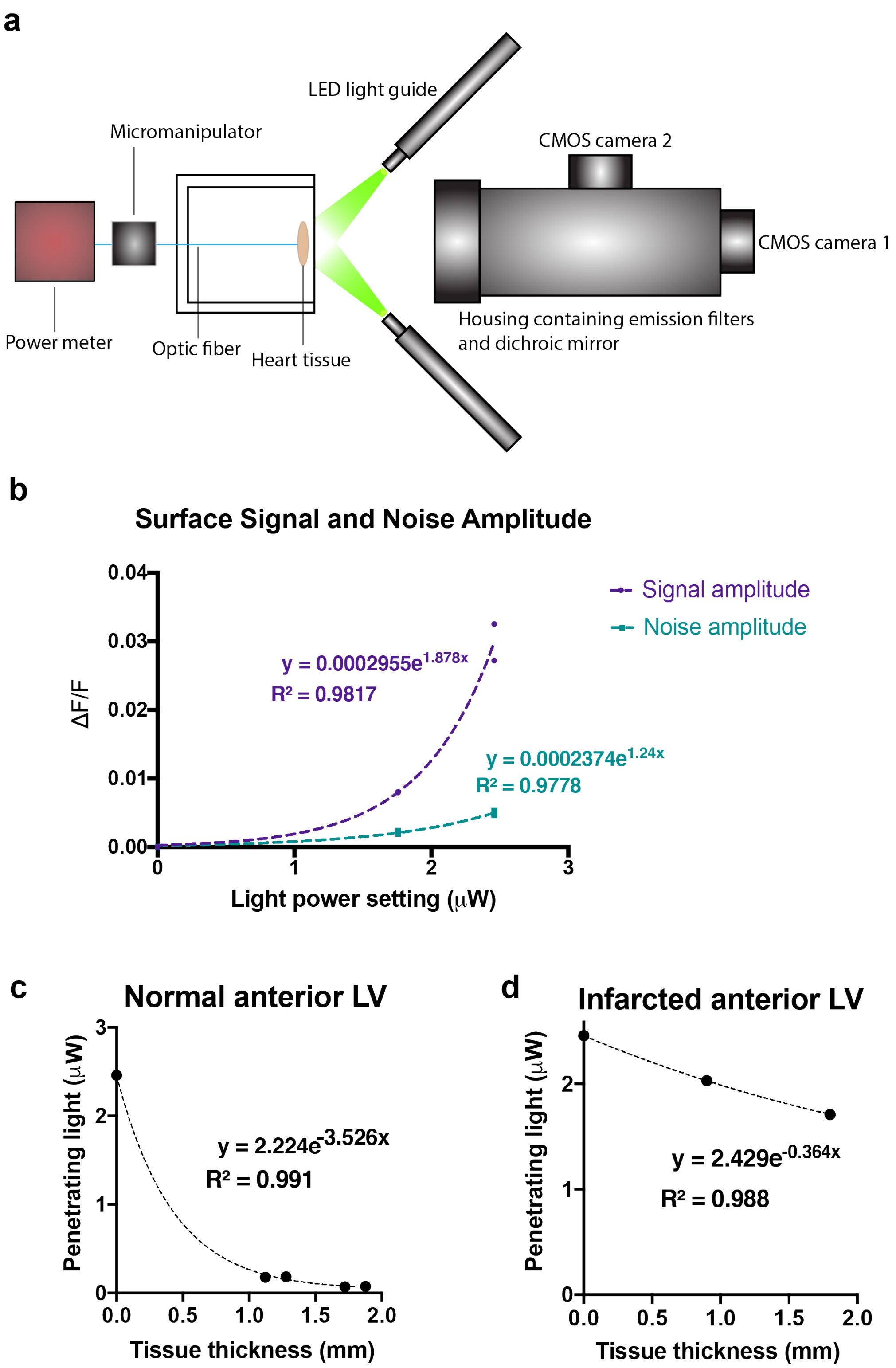

Supplemental Figure 7. Light penetration measurements to calculate epicardial shell depth. To ensure structural image features were extracted from the same depth as optical action potential (AP) signals, we empirically determined the depth of AP signals from our optical mapping system by measuring light penetration through myocardial tissue slices of varying

thicknesses **(a)**. First, we plotted the relationship of different excitation light power settings to the detected signal and noise amplitude from 2 hearts **(b)**. With this plot, we used exponential regression to determine the excitation light power at which the resulting signal amplitude is equal to the noise amplitude generated by the maximal light power setting. Next, we measured the penetrating light power through normal **(c)** and infarcted **(d)** anterior LV tissue slices of different thicknesses, which displayed expected exponential decay curves per Beer's Law. Finally, we used exponential regression to calculate the tissue depth at which the light power calculated from **(b)** occurs. This was approximately 100μm deep, when correction is applied for tissue shrinkage during modified immunolabeling-enabled 3-dimensional imaging of solvent-cleared organs (iDISCO). Since this is the depth where the penetrating light power can only generate a signal amplitude equal to the noise amplitude at the surface, we defined this as our structural shell depth.

Supplemental Movie 1. Dense scar with surviving myocytes mostly on endocardial side. Cleared heart showing 3-dimensional dense scar structure. In addition to excluding structural data from dense scar regions, we also excluded optical mapping data, as most of the surviving myocytes in this region were on the endocardial surface and thus not contiguous with the epicardial shell we used for our quantitative analyses. TH = tyrosine hydroxylase, NFH = neurofilament heavy (1:200, Sigma-Aldrich, AB5539).

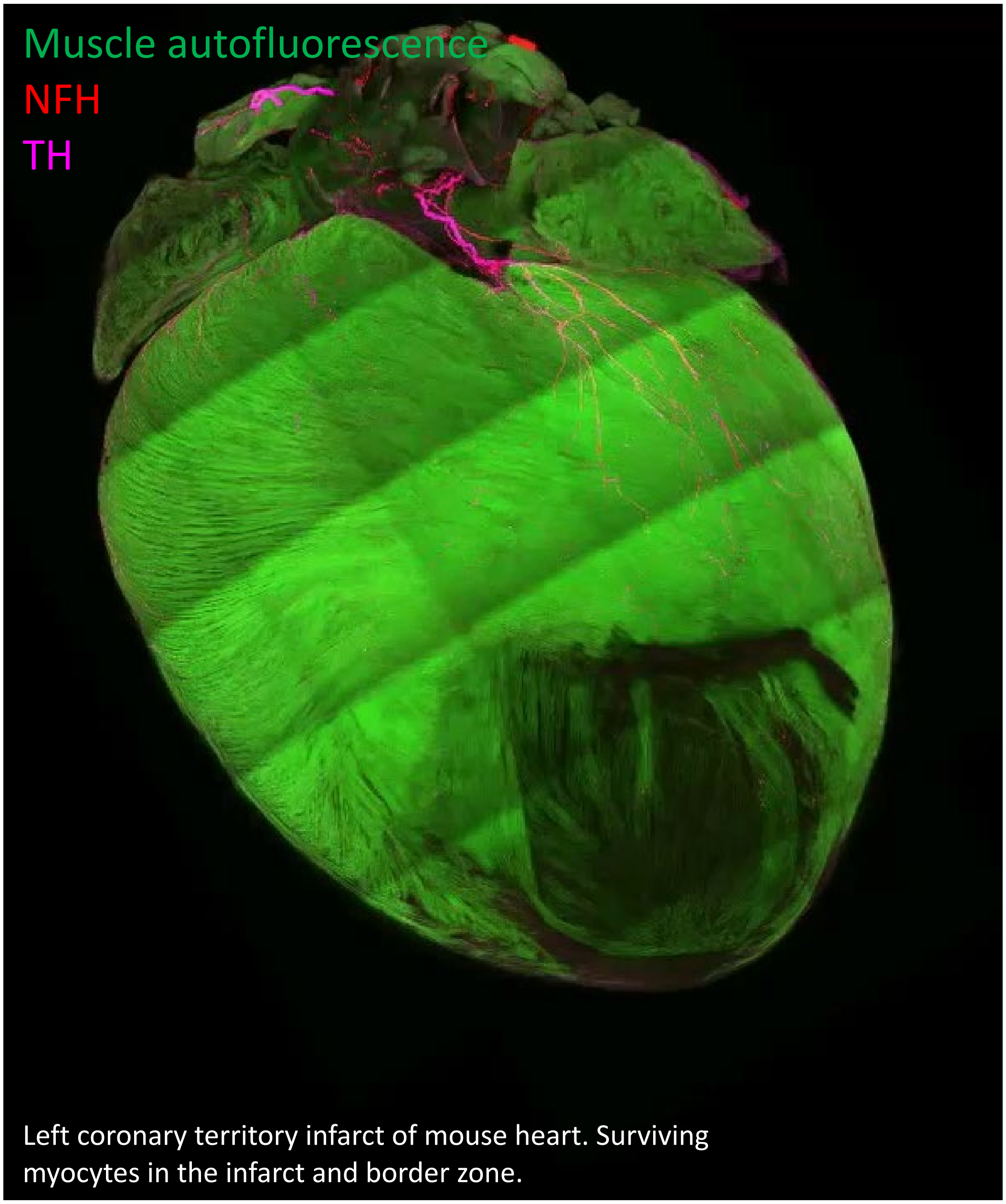

Supplement: Supplemental data [file jciinsight-7-153913-s177.pdf]
